# Atomistic Insights into Solid-State Phase Transition Mechanisms of P2-Type Layered Mn Oxides for High-Energy Na-Ion Battery Cathodes

**DOI:** 10.1021/acsenergylett.4c03335

**Published:** 2025-02-06

**Authors:** Aniello Langella, Arianna Massaro, Ana B. Muñoz-García, Michele Pavone

**Affiliations:** † Department of Chemical Sciences, University of Naples Federico II, Complesso Univ. Monte Sant’Angelo Via Cintia 21, Naples 80126, Italy; ‡ National Interuniversity Consortium of Materials Science and Technology - Reference Center for Electrochemical Energy Storage (INSTM-GISEL), Via G. Giusti 9, Firenze 50121, Italy; § Department of Physics “E. Pancini”, University of Naples Federico II, Complesso Univ. Monte Sant’Angelo Via Cintia 21, Naples 80126, Italy

## Abstract

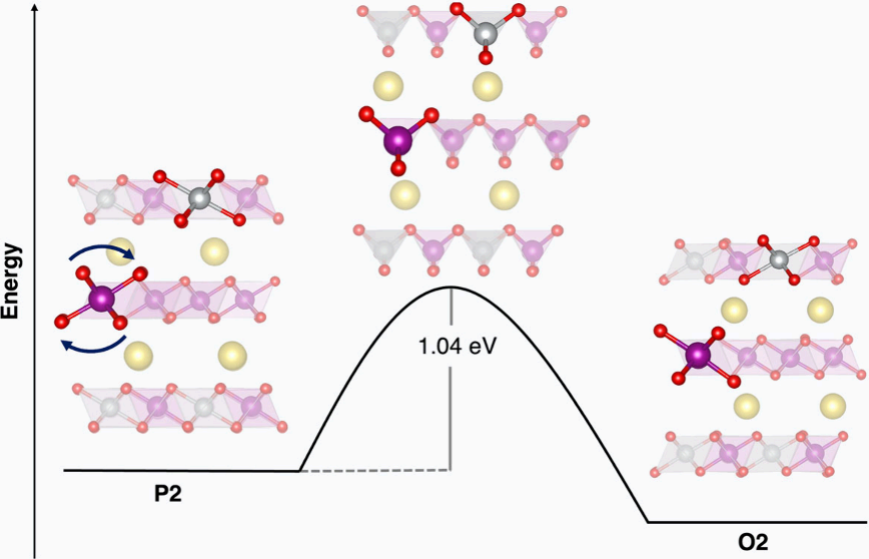

Mn-based layered oxides hold great promise as high-energy, cost-effective cathodes for sodium-ion batteries (NIBs), but repetitive Na^+^ cycling induces harmful phase transitions. Understanding these mechanisms is essential for designing better performing NIB cathodes. Applying density functional theory (DFT) and variable cell-nudged elastic band (VC-NEB) calculations, we provide atomistic insights into phase transformation pathways and energy barriers in P2-Na_*x*_MnO_2_ material and its Ni-doped variant. We reveal the key P2-to-OP4/O2 and P2-to-P2′ transitions that occur across various sodiation levels, involving substantial rearrangements around the transition metal sites, with tetrahedral transition states accountable for energy barriers. Our analysis of bond length and angle distortions highlights that shear deformations are pivotal in triggering P-to-O gliding at low sodium levels. Based on these insights, our structural distortion metrics offer a straightforward and computationally efficient descriptor to evaluate structural integrity for these layered oxides, enabling the design of NIBs with improved stability and extended lifespan.

Integrating renewable energy into the electric grid demands large-scale energy storage systems (ESSs) to buffer supply fluctuations and manage peak energy demands.^1^ Among available ESSs, lithium-ion batteries (LIBs) excel for their flexibility, high efficiency, and low maintenance,^2,3^ making them widely adopted in portable devices and electric vehicles and for significant reduction of dependence on fossil fuels.^4^ However, the limited and uneven distribution of lithium resources,^5^ combined with the rising demand from the electric mobility sector, may drive up lithium costs and deplete its natural reserves.^6^ Sodium, the fourth most abundant element on Earth, offers a more sustainable alternatives to LIBs, offering a viable option for large-scale ESSs.^4,7,8^ Although research on Na-ion batteries (NIBs) began alongside LIBs in the 1970s,^9^ major breakthroughs have been achieved only in the past two decades. Yet, greater advances concern anode materials,^4,10−13^ while key challenges related to the development of high-energy, stable cathode materials still prevent the wide deployment of NIBs in the market.^4,14,15^

Early studies on Na-based cathodes focused on layered transition metal oxides (TMOs), which resemble lithium analogues but display different electrochemical behavior and more intricated polymorphism.^16−18^ Indeed, compared to lithium counterparts, the larger ionic radius of Na^+^ exacerbates structural instabilities of cathodes during battery operations, driving performance issues.^19−21^ Various TMOs, including those based on manganese, nickel, vanadium, and iron, have been explored,^16,13,22^ with phases categorized as P2 or O3 depending on the stacking of oxygen atoms and sodium ion coordination sites (“O” for octahedral and “P” for prismatic).^19,23,24^ P2-type materials generally offer better ion diffusion and electrochemical performance,^19,25^ and O3 phases tend to provide higher capacities and sustained voltage during cycling.^19,26,27^ Both, however, suffer from severe structural charges during repeated charge/discharge cycles^19,28^ (*i.e.*, cyclic extraction/insertion of Na^+^ across the TMO_2_ layers), leading to capacity loss and mechanical degradation.^28^ Two main phase transitions are observed in these materials: O3-to-P3, involving complex stacking changes during desodiation even at a low state of charge (SoC)^19,28^ and P2-to-OP4/O2, which concerns the TMO_2_ layers gliding at high SoC and leads to a significant (over 20%) volume variation.^28^

Active research focuses on improving phase stability by employing strategies such as doping TMs, creating TM vacancies, applying protective surface coatings, optimizing synthesis methods, and developing P2/O3 intergrowth composites to exploit the benefits of both phases.^29−41^ While these strategies have improved electrochemical performance, the underlying mechanisms driving the solid-state phase transitions still remain elusive. Unveiling the atomistic features of these mechanisms could provide new fundamental knowledge for deriving novel rational design principles toward development of improved NIB cathodes with greater stability and efficiency.

Mn-rich cathodes are particularly appealing for large-scale production due to Mn high abundance and low costs of raw materials.^14,17,18^ During sodium removal upon charging, Jahn–Teller (JT) active Mn^3+^ partially oxidizes to Jahn–Teller inactive Mn^4+^, causing complex distortions in MnO_6_ octahedra.^42^ These distortions propagate beyond individual octahedra, resulting in a macroscopic symmetry reduction.^42−44^ At the beginning of the charging process, NMO adopts a *P*6_3_/*mmc* space group^38,42^ (center structure in Figure 1a); but as the sodium content (*x*_Na_) drops below 0.4, an irreversible phase transition shifts the P2 structure to OP4^38^ (left structure in Figure 1a), altering the sodium layer from purely prismatic to a mixed prismatic-octahedral arrangement. With Na reinsertion up to *x*_Na_ > 0.7, a P2′ phase with a *Cmcm* space group emerges^38^ (right structure in Figure 1a). Nickel doping is frequently adopted in TMO production, as the Ni^2+^/Ni^3+^/Ni^4+^ electroactive redox couples can sustain high operating voltages, enabling full Na^+^ exchange across a 2.0–4.5 V window^45−47^ and unlocking extra capacity from the activated oxygen sublattice above 4.1 V.^48,49^ On including nickel in the layered TMO, in P2-Na_*x*_Ni_0.25_Mn_0.75_O_2_ (NNMO), however, phase transitions also occur (Figure 1b): while the undoped NMO undergoes a P2-to-OP4 transition around 3.5 V,^50−52^ an NNMO solid-state phase transition occurs at approximately 4.1 V,^48^ corresponding to *x*_Na_ < 0.25.^33^ Reducing the cutoff voltage to 4.1 V could enhance NNMO cycling stability and structural integrity, but this would lower capacity by over 40%, limiting Na^+^ extraction and diminishing oxide redox activity.^53^

**Figure 1:**
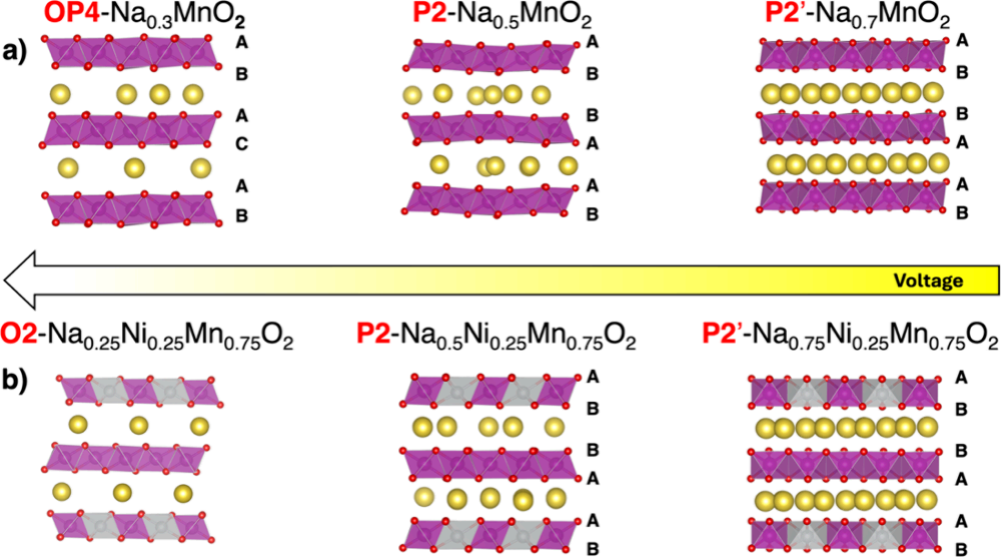
Schematic diagrams of (a) Na_*x*_MnO_2_ (NMO) and (b) Ni-doped Na_*x*_MnO_2_ (NNMO) phase evolution upon battery functioning. View along the *y* axis. Atoms are represented as spheres, and TMO_6_ (TM = Mn, Ni) moieties are represented with polyhedra. Color code: Na, yellow; Mn, violet; O, red; Ni, gray.

Recently, we employed first-principles analysis to examine the phase stability of NMO, focusing on the structural and electronic characteristics of various phases (P2, P2′, and OP4) across different sodium levels, corresponding to varying SoC during cycling.^42,54^ We identified the cooperative Jahn–Teller effect (CJTE) and Na^+^/vacancy ordering as primary factors impacting phase stability, which could serve as thermodynamic triggers for undesired phase transitions.^42^

Starting from these promising results, here, we address the phase evolution of P2-NMO and its Ni-doped counterpart, NNMO, with a detailed focus on the atomistic mechanisms and energy barriers for these solid-state transitions. Using the variable cell-nudged elastic band (VC-NEB) method, we investigate the kinetic features of P2-to-P2′ and P2-to-OP4/O2 solid-state conversions. To this end, we apply state-of-the-art Density Functional Theory (DFT), which provides a robust framework to explore structure–property relationships in solid-state functional materials. Recent studies on transition metal oxides (TMOs) have used DFT-based methods to evaluate promising cathode candidates for future batteries, focusing on dynamical phase stability^55,56^ and electronic structure evolution upon Na removal.^54,57,58^ Here, we use the PBE+U(D3BJ) level of theory to analyze the structural and electronic characteristics of P2-, P2′-, OP4-, and O2- phases for NMO and NNMO materials at different Na contents, simulating the cathode desodiation (*i.e.*, the charging process). We first map the transition pathways and energetics to reveal the atomistic mechanisms at both high and low degrees of sodiation. Subsequently, we investigate structural distortions around the transition-metal-centered octahedra, driven by the differing Jahn–Teller activity of oxidized TM centers upon Na^+^ removal. By dissecting bond-length and bond-angle distortions, we directly link these structural changes to the relative phase stability and the transition pathways, offering valuable insights into the solid-state transformations in these advanced NIB cathode materials.

While initial and final states of the P2-to-P2′ and P2-to-OP4/O2 transitions in both NMO and NNMO are well documented,^19,38^ the transformation pathways themselves remain largely unexplored. To uncover the mechanisms and associated barriers of these complex solid-state transitions, we employ the variable cell-nudged elastic band (VC-NEB) method, as implemented in the USPEX code.^59,60^ Unlike the conventional NEB approach, which constrains cell dimensions to those of the fixed end points, the VC-NEB method permits simultaneous variation of both cell parameters and atomic coordinates along the search for the minimum energy path (MEP). We model the P2-to-P2′ and P2-to-OP4/O2 transitions from P2-NMO and P2-NNMO using the *pmpath* tool,^61^ an algorithm capable of generating transition pathways based on symmetry operations and cell mapping. Each pathway is divided into 20 intermediate steps (images) to capture the stepwise structural evolution. To the best of our knowledge, this study represents the first comprehensive application of VC-NEB to this class of material, providing an unprecedented view of the atomistic mechanisms behind these critical phase transitions. Details on the validation of our methodology can be found in Figure S1 in the Supporting Information (SI), where we demonstrate that, for NMO, the P2-to-P2′ transition is kinetically favored over the P2-to-OP4 one for high sodium content, while the reverse holds true at low Na levels. This outcome aligns with experimental findings where P2′ and OP4 phases emerge at high and low Na concentrations, respectively, from the initial P2 phase.^38,42^Figure 2 displays the computed barriers for the key transitions for NMO and NNMO at their critical Na contents. For NMO, P2-to-P2′ is evaluated at *x*_Na_ = 0.75, while P2-to-OP4 is considered at *x*_Na_ = 0.375 (Figure 2a). For NNMO, the barriers are calculated for the P2-to-P2′ transition at *x*_Na_ = 0.75 and the P2-to-O2 transition at *x*_Na_ = 0.125 (Figure 2b). NNMO shows a significantly higher energy barrier for the P2-to-P2′ transition compared to NMO (1.70 eV *vs* 1.24 eV), indicating enhanced structural stability at high sodium content. Conversely, the P2-to-O2 transition at low sodium content in NNMO exhibits a reltively low energy barrier of 1.04 eV, indicating an easier phase conversion process and aligning with experimental observations of facile phase transitions occurring at high voltage.^48^ For completeness, we computed the barrier for the P2-to-O2 transition in NNMO at *x*_Na_ = 0.375, the critical Na content for phase transition in NMO. As shown in Figure S1 of the SI, this transition exhibits a significantly higher barrier of 2.9 eV, suggesting that the P-to-O conversion in the Ni-doped material remains largely suppressed at this SoC and is only triggered when the material is pushed at high voltages (very low sodium content).

**Figure 2:**
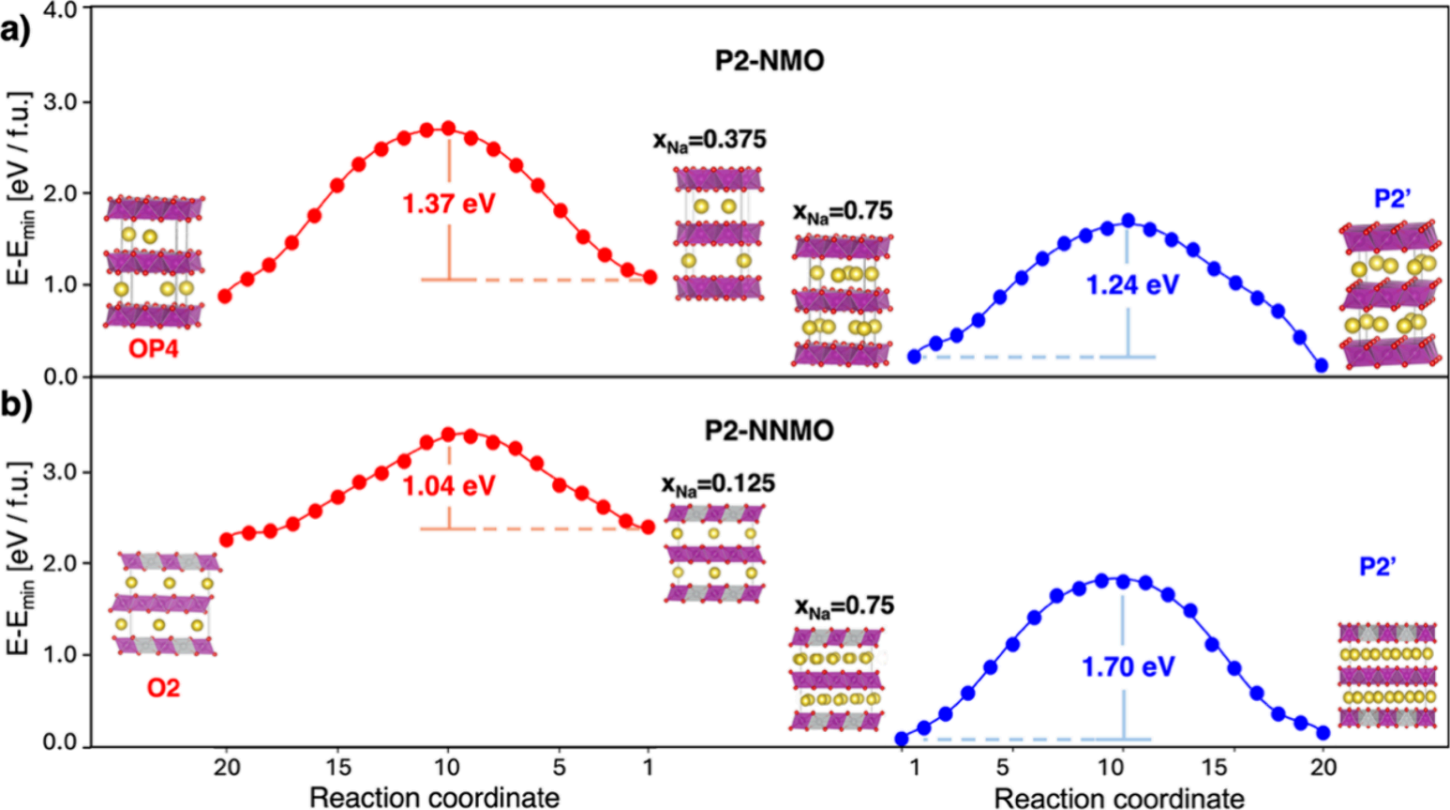
Energy profiles for phase transitions at the critical Na content *x*_Na_ in (a) NMO and (b) NNMO. P2-to-P2′ and P2-to-O transitions and corresponding barriers are plotted in blue and red, respectively.

To unveil the origin of these energy barriers, we analyze the atomic-level structural rearrangements along the reaction coordinate, as schematically depicted in Figure 3. Two primary atomic motions emerge as driving modes of the phase transitions: (i) the shifting of TMO_6_ units within the *ab*-plane, involving a combined rotation and translation of the octahedral units (Figure 3a); (ii) the gliding of distinct TMO_2_ layers, which entails both a mirroring and translation of the layers (Figure 3b,c). The first motion primarily affects the coordination environment around the transition metal, while the second leads to substantial stacking rearrangements across the lattice. Given that the OP4 and O2 phases share the same space group and differ only in Na site occupancies (see Figure S3), we analyze the P2-to-OP4 transition in NMO and the and P2-to-O2 transitions in NNMO within a unified framework, collectively referring to them as P2-to-O transitions in this section of the manuscript.

**Figure 3:**
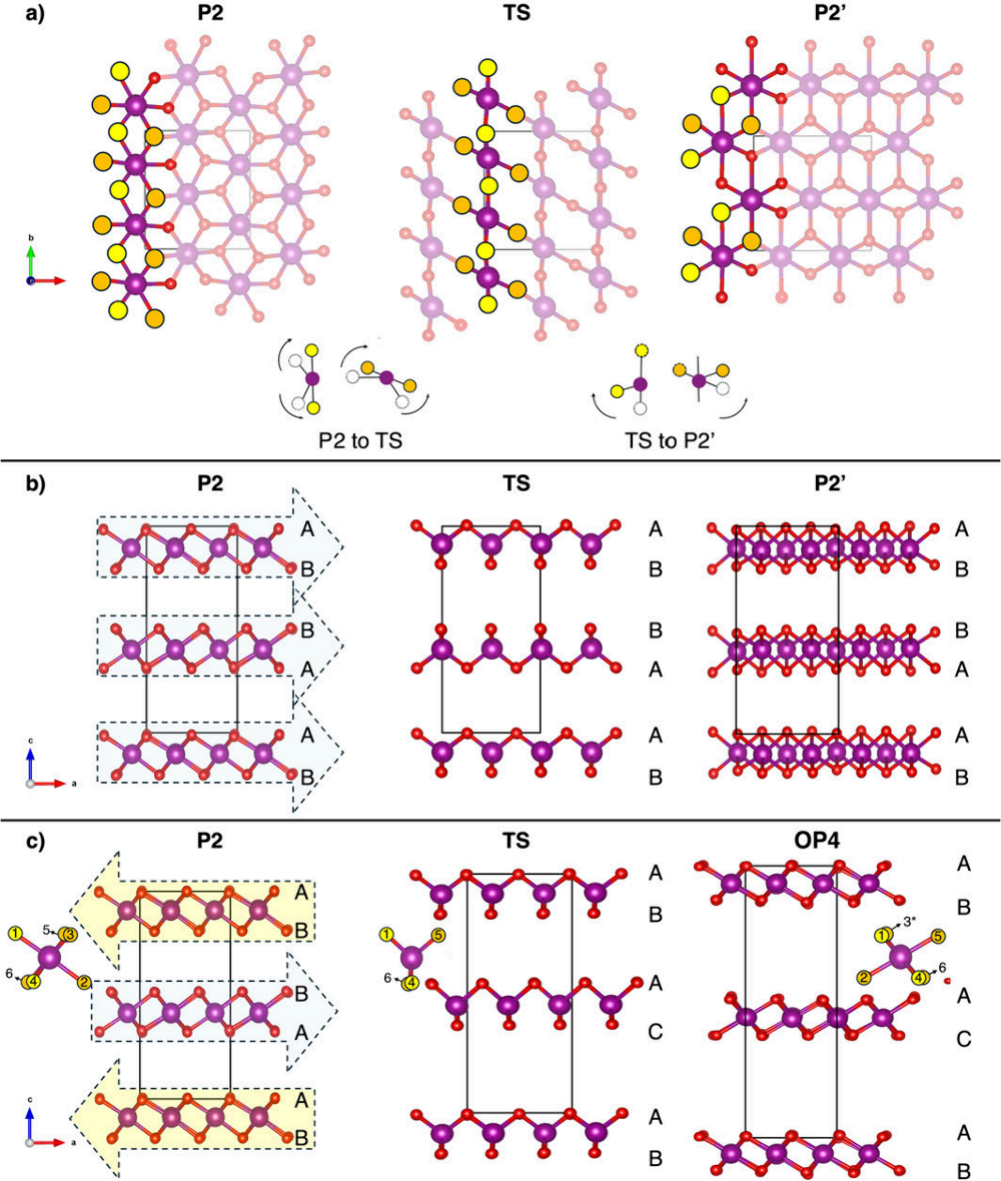
(a) Schematic representation of the proposed mechanism for the P2-to-P2′ transition, highlighting the shift of TMO_6_ units within the *ab*-plane. A single row of atoms is rendered fully opaque to emphasize the shift, with yellow and orange circles marking the oxygen ligands involved and their respective movements. Details of the octahedral axis rotation associated with this mechanism is displayed. Dotted and colored circles depict the initial and final positions, respectively. (b) and (c) Schematic representation of the TMO_2_ sheets gliding as the proposed mechanisms for the P2-to-P2′ and P2-to-OP4 transitions, respectively. Dotted arrows indicate translation directions. The Mn–O bond configurations for each structure are shown, with yellow and orange circles highlighting the oxygen ligands involved and the rotation along the P–O glide. Color code as in Figure 1.

A closer look at the P2-to-P2′ transition mechanism reveals that the in-plane shift prompts a significant structural reorganization around the TM center, resulting in tetrahedrally coordinated transition states (Figure 3a,b). This motion pattern remains consistent across all layers with the TMO_2_ sheets coherently translating in the same direction without gliding. Consequently, the ABBAAB oxygen layer stacking is preserved throughout the transition. In contrast, the P2-to-O transition (Figure 3c and Figure S4) involves the formation of a similar tetrahedral-like transition state, achieved not through in-plane, but purely *via* the gliding and π/3 rotation^19^ of alternating TMO_2_ layers moving in opposite directions, which alters the oxygen atom stacking to ABACAB. Rotations around TM centers for this transition are highlighted in the insets of Figure 3c and in Figure S5. The presence of tetrahedrally coordinated TM at the transition states of both the P2-to-P2′ and P-to-O transitions aligns well with experimental findings. Indeed, extended X-ray absorption fine structure (EXAFS) analyses have reported variations in the transition metal coordination number during battery charging, supporting the hypothesis of a structural reconfiguration.^49^ To verify the accuracy and robustness of the tetrahedral transition states derived from the VC-NEB approach, we have compared the structures obtained using the *pmpath*(61) tool with those derived from the dimer method optimization,^62,63^ which is specifically optimized for transition states investigation, for the TS configurations found along both the P2-to-P2′ transition at *x*_Na_ = 0.75 and the P2-to-OP4 transition at *x*_Na_ = 0.375 in P2-NMO. As shown in Figure S6, the comparison demonstrates an excellent match between the TS structures, with only a minor discrepancy involving one 5-fold-coordinated Mn atom. Despite the inherent complexity in identifying metastable structures, these results confirm the reliability of *pmpath* in capturing the intricacies of phase transitions and its ability to outperform traditional interpolation-based pathway methods.^63^

Overall, the main distinction between P2-to-P2′ and P2-to-O transitions lies in the alignment of the oxygen atoms at the interlayer region hosting Na ions. In both P2 and P2′ structures, the TMO_6_ layers remain superimposable, while in the O-type phases this alignment is disrupted, enabling the formation of the characteristic octahedral sites of these structures.

This analysis of the transition mechanisms, combined with considerations on inter- and intralayer interactions, support the VC-NEB findings: at low voltage, high sodium content effectively shields the O^2–^···O^2–^ interlayer repulsion, minimizing the need for TMO_2_ layers gliding and preserving their superimposability. This is also the reason behind the unfavorable P2-to-O transitions compared to P2-to-P2′ at high *x*_Na_. Conversely, at low *x*_Na_, increased O^2–^···O^2–^ repulsion and sodium rearrangements to more stable *displaced* sites^42^ favor the P2-to-OP4 transition over the P2-to-P2′ one. Electronic structure analysis (Figures S7–S10 in the SI) uncovers that the P2–O glides are coupled to negligible changes in the oxidation of transition metals, with Mn^4+^ and Ni^4+^/Ni^3+^ being retained across this transition in both NMO and NNMO.^42,57^ More pronounced variations take place along the P2-to-P2′ transition, with the P2′ phase containing a higher concentration of Mn^3+^ and Ni^3+^. As JT active centers, Mn^3+^ and Ni^3+^ sites are responsible for structural distortions^64^ and will be highlighted later as key drivers toward the low-voltage transition.

On the other hand, mechanistic insights on the P-to-O glides require detailed structural analysis, which can be better understood by examining the distortion modes in both NMO and NNMO. To this end, we recently analyzed the structural evolution of P2-NMO structures upon charge (*i.e.*, *x*_Na_ = 0.72, 0.59, 0.47 and 0.34) in terms of the average octahedral distortion, *D*_OCT_.^42,56^ In an ideal octahedral geometry, *D*_OCT_ would be zero, as the six ligands are symmetrically coordinated to the TM center, and we further decompose *D*_OCT_ to identify the subtle features of possible distortions. These may stem from bond-length variations, often driven by the JT effect, and/or from bond-angle deviations, each contributing differently to the overall *D*_OCT_ value. To capture these contributions, we calculated Van Vleck modes,^65^ which account for all the possible ligands (oxide ions) displacements from their ideal positions. Of the six relevant modes (Figure S11 in the SI), the Q2 and Q3 ones share the same symmetry with the e_g_ orbitals^65^ and are thus sensitive to JT-driven bond variations, while the Q4, Q5, and Q6 modes describe angular deformations.^66,67^ We analyzed these two primary contributions (bond-length and bond-angle distortions) by examining the average magnitude of the distortion (ρ_0_) and the fraction parameter (η) proposed by Nagle-Cocco and Dutton.^67^ Additional details on the distortion parameters and their formulas are provided in Figure S12 and in the Distortion parameters section of the SI. In NMO (Figure 4, first column), the *D*_OCT_ analysis revealed a distinct U-shaped trend, reaching a minimum at *x*_Na_ = 0.47, a composition with a unique Mn^3+^/Mn^4+^ distribution and the presence of a cooperative Jahn–Teller effect (CJTE).^43,68^ For the highest degree of sodiation explored (*x*_Na_ = 0.72), NMO presents a *D*_OCT_ around 6.8%, and as Na^+^ ions are removed, *D*_OCT_ initially decreases, reflecting the partial oxidation of Mn^3+^ to Mn^4+^. Upon reaching a minimum of ∼5.4% for *x*_Na_ = 0.47, *D*_OCT_ increases, indicating that further distortions emerge as more Na^+^ is extracted (Figure 4, first column, first panel). We have computed *D*_OCT_ also for the P2-NNMO system at *x*_Na_ = 0.75, 0.50, 0.375, 0.25, and 0.125 so as to account for the whole sodiation range and to maintain consistency with other computational studies (Figure 4, second column, first panel).^33^ The minimum value of *D*_OCT_ in NNMO is shifted to *x*_Na_ = 0.25, where a peculiar cation distribution is present within the *ab*-plane (see Figure S13): similar to other layered oxide structures,^42,43^ a distinct stripe-like arrangement appears, where alternating layers of pure Mn^4+^ and mixed Mn/Ni layers with different oxidation states are observed. This distinctive pattern contributes to a more uniform distribution of Mn/Ni–O bond distances at *x*_Na_ = 0.25 (see the pair distribution function, PDF, reported in Figure S14). The computed ρ_0_ for P2-NMO, shown as a function of *x*_Na_ (Figure 4, first column, central panel), follows the expected trend in Mn oxidation upon charging: as Mn^3+^ is progressively oxidized to Mn^4+^, ρ_0_ decreases as octahedra are approaching their ideal configurations. The higher the ρ_0_ value, the larger the bond-length distortion contribution, aligning with a larger presence of JT-active centers and suggesting a pronounced tendency toward the P2-to-P2′ transition at low voltages. In NNMO, the ρ_0_ trends for Mn and Ni centers (Figure 4, second column, central plot) highlight the subtle balance required for optimal doping: as in NMO, ρ_0_ of Mn shows a steady decrease as Mn^3+^ oxidizes to Mn^4+^. Conversely, the oxidation of Ni^2+^ to Ni^3+^ (and eventually Ni^4+^) raises ρ_0_, driven by the JT activity of Ni^3+^, which induces significant bond-length distortions. The divergent ρ_0_ trends for Mn and Ni imply that, while higher nickel doping could enhance electrochemical performance, it might also compromise structural stability by reversing the trend of the weighted averaged ρ_0_ (black dotted line), which for low Ni amounts follows the Mn trend, and accelerating the undesired P-to-O phase transitions. Nevertheless, NNMO exhibits structure-stabilizing, smaller ρ_0_ absolute values compared to NMO in spite of the presence of two JT active ions (Mn^3+^ and Ni^3+^). This advantage could be attributed to Ni^3+^–O bond lengths, which fall between those of Mn^4+^–O and Mn^3+^–O (as shown in the PDF in Figure S13). Furthermore, Ni doping is known to enhance the covalent nature of the TM–O bond, thereby helping to mitigate the interlayer shifts in other Mn-based layered oxides.^64,69^ As a matter of fact, the P2-to-P2′ transition in NNMO shows a higher energy barrier as discussed previously (Figure 2).

**Figure 4:**
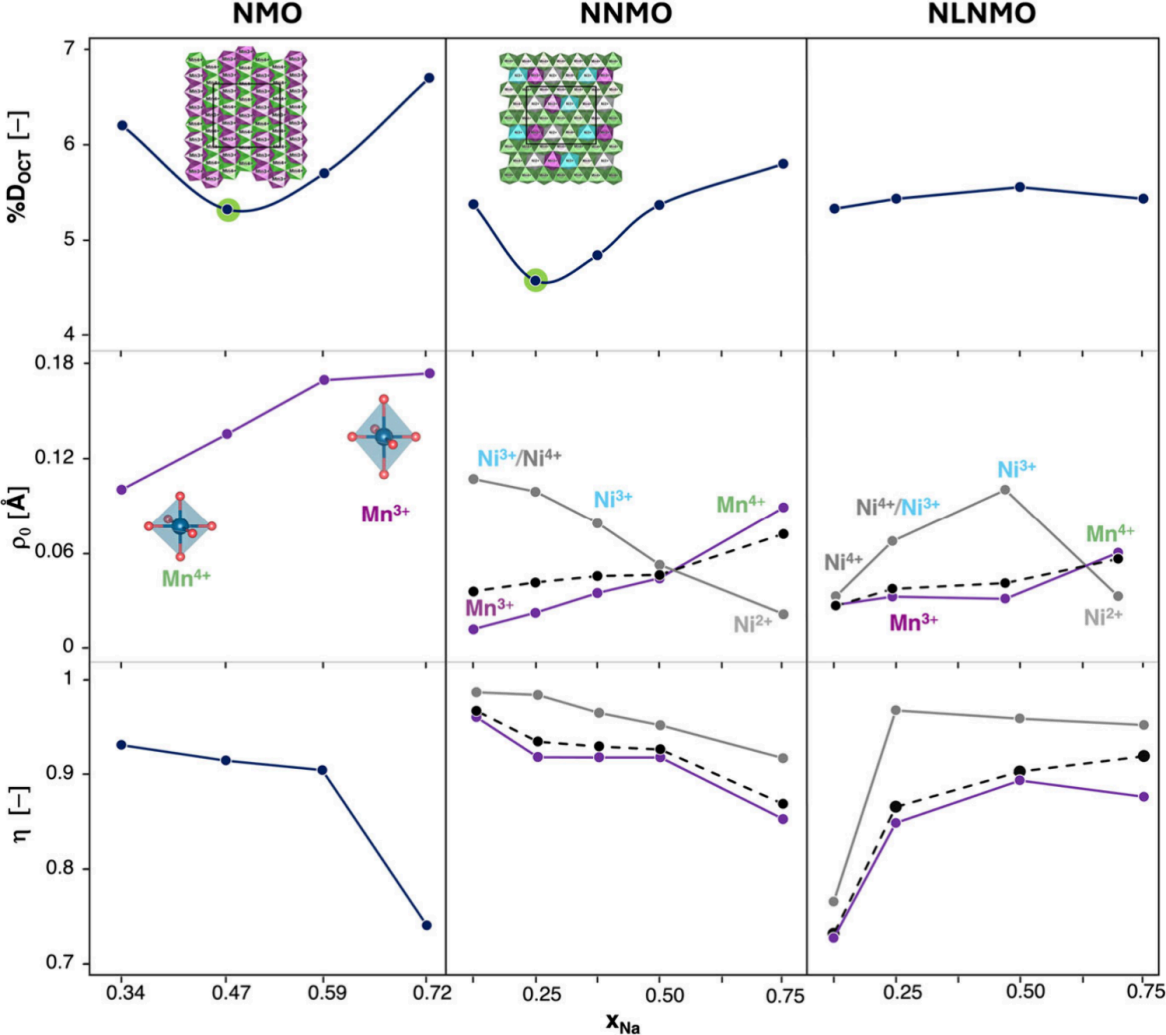
Structural distortion parameters plotted as a function of sodium content, *x*_Na_, in (left) P2-NMO, (middle) P2-NNMO, and (right) P2-NLNMO. This first row shows the octahedral distortion, %*D*_oct,_ as a function of *x*_Na_ for the three materials. The second and the third rows present the magnitude of the distortion, ρ_0_, and fraction parameter, η, respectively. Insets are as follows. (first row) Cation distributions in the *ab*-plane with O and Na atoms removed for clarity. Color code: Mn^3+^-centered octahedra, violet; Mn^4+^-centered octahedra, green; Ni^2+^-centered octahedra, gray; Ni^3+^-centered, turquoise. (Second row) schematic of an ideal Mn^4+^O_6_ octahedron and axially elongated Mn^3+^O_6_ one.

Regarding angular distortions, bond-angle-dependent modes Q4–Q6 are related to the displacements of the ions from their ideal positions due to octahedral tilting.^66^ For NMO, the trends of the three modes are opposite those of ρ_0_, as shown in Figure S15a. This suggests that the *D*_OCT_ trend observed in NMO (Figure 4, first column, first panel) arises from a complex interplay between bond-length distortions (ρ_0_, Figure 4, first column, central panel) and such bond-angle variations (Q4 to Q6 modes, Figure S15a). Qualitatively, at high sodium content (0.47 < *x*_Na_ < 0.72), bond-length distortions dominate the structural changes, leading to a decrease in *D*_OCT_ that mirrors the ρ_0_ trend. Below *x*_Na_ = 0.47, reduced electrostatic screening of the O^2–^···O^2–^ repulsions leads to pronounced octahedra tilting, which drives the observed increase in *D*_OCT_.^70^ The fraction parameter, η (Figure 4, first column, third panel), increases continuously as *x*_Na_ decreases, indicating a growing contribution from angular distortions upon desodiation (Figure S15a). Notably, η remains below 1, indicating the coexistence of both shear and antishear distortion modes. The proposed reaction pathway supports these findings, with the tetrahedral transition state (TS) arising from the interplay of these modes: shear distortions that facilitate Mn–O bond cleavage, while antishear modes stabilize the TS tetrahedral configuration (Figure 3). Such interplay between bond-length and bond-angle distortions of P2-NMO during sodium extraction highlights again the crucial role of the Mn^3+^/Mn^4+^ distribution and sodium ion reorganization in determining the structural evolution of the material. The combined effects of these distortions are essential for the transition from the P2 to O2/OP4 stacking, where oxygen sublattice reconfiguration is achieved *via* a series of simple rotations. Compared to NMO, P2-NNMO exhibits generally lower bond-angle distortions (Figure 4, second column, third panel, and Figure S15a,b), with a dominant shear-like character, as indicated by η values closer to 1 at lower sodium content. As η approaches 1, the energy barrier for the MnO_2_-layer gliding (Figure 2b and Figure S2) tends to decrease, facilitating the phase transition.

Preventing TMO_2_-layer gliding at high voltages requires strategies that not only target the reduction of angular distortions (a challenging task due to the strong interlayer repulsion following sodium removal) but also aim at minimizing shear distortions as much as possible. Effective approaches aiming at preserving the cathode structural integrity under elevated voltages include tailored metal doping to strengthen TM–O bonds,^71^ introduction of TM vacancies, and incorporation of inert elements,^71^ all of which acting as inhibitors of shear-induced transitions. Notably, as demonstrated by Massaro *et al.*,^32,33,36^ increasing TM–O covalency is not only an effective strategy but also computationally straightforward to evaluate. For example, Li-doped P2-NNMO systems have demonstrated resilience to phase transitions at high voltages.^72^ As a proof of concept to assess whether enhancing structural stability and preventing detrimental P–O glide depend on minimizing shear distortions, we extend our analysis to P2-Na_*x*_Ni_0.125_Li_0.125_Mn_0.75_O_2_ (NNLMO) at *x*_Na_ = 0.75, 0.50, 0.25, and 0.125 (Figure S16). P2-NNLMO (Figure 4, third column, first panel) exhibits a much more constant *D*_OCT_ trend as *x*_Na_ decreases compared to P2-NMO and P2-NNMO. This stability is mirrored by a steady reduction in distortion magnitude (ρ_0_) during desodiation (Figure 4, third column, central panel, dotted black line), primarily driven by the diminished Jahn–Teller (JT) effect in Mn (purple line). The non-monotonic behavior observed for Ni (gray line) is primarily attributed to the exclusive presence of Ni^4+^ at the end of charging.

Since distortions are evaluated within the TMO_6_ layers, nickel in anti-site positions is excluded from the calculation of distortion parameters. Importantly, the lower Ni concentration in NNLMO compared to NNMO minimizes these anti-site defects, enabling a more stable behavior. This stability is reflected in the slight reduction in ρ_0_, suggesting enhanced stability and a minimized risk of interlayer shifts. The initial increase in Q4, Q5, and Q6 modes upon desodiation accounts for the small rise in *D*_OCT_ at early desodiation stages (Figure S15c). At a lower sodium content (*x*_Na_ = 0.25), however, these modes begin to decrease, aligning with the overall trend in *D*_OCT_. Furthermore, the behavior of η (Figure 4, third column, third panel) reinforces the crucial role of shear modes in the phase transition mechanism: despite fluctuations during charging, η never reaches 1 and actually decreases at high voltages, indicating a diminishing contribution from pure shear modes. This behavior is likely influenced by the presence of positive cations other than sodium at the TMO_6_ interlayer region, which inhibit these shear distortions. Notably, the energy barrier for the P2-to-O2 transition in NNMO decreases as sodium content drops (2.9 eV at *x*_Na_ = 0.375, 2.0 eV at *x*_Na_ = 0.25, and 1.04 eV at *x*_Na_ = 0.125), corresponding to an increasing fraction parameter. This confirms that the P-to-O gliding transition is primarily driven by shear modes.

Noteworthy, for NNMO the anionic redox activity is also a well-studied phenomenon, because of its key contribution to enhanced energy density, but it typically involves oxygen release, which can lead to structural deterioration.^19,57^ Strategies to exploit anionic activity without triggering molecular oxygen release have been explored,^57^ but these are beyond the focus of the present work. Other challenges, such as cation migration to interlayer sites and the reversibility of these processes, remain critical areas for further investigations. Despite these additional complexities, our structural analysis still provides a decisive conclusion: mitigating both Jahn–Teller (JT)-induced bond-length distortions and shear-induced deformations of TM-centered octahedra in Na-based layered TMOs can directly address two key issues. Specifically, reducing JT distortions prevents interlayer shifts that drive the P2-to-P2′ transition during resodiation, while minimizing shear-induced deformations inhibits the TMO_2_ gliding responsible for P-to-O phase conversion at high voltages. The trends observed in Van Vleck-derived descriptors, supported by experimental data, strongly suggest that controlling the η/ρ_0_ ratio can enhance resistance of layered TMOs to P–O glides and P2–P2′ shifts. Fine-tuning shear modes and JT activity emerges as a pivotal strategy for optimizing these materials for practical applications, achievable through tailored modifications such as metal doping or defect engineering at the cathode. The fundamental principle behind these modifications is to increase the structural rigidity of the system, thereby preventing glide phenomena. This can be accomplished by enhancing TM–O bond covalency (*e.g.*, through the partial substitution of TM with d^0^ elements or alkali metals)^33,71^ or reducing interlayer repulsions *via* strategic metal substitutions. For instance, K-doping or codoping at sodium sites offers a promising approach.^38,71^ By occupying sodium sites without participating in redox processes and thus remaining in the structure throughout charge–discharge cycles, potassium may be very effective in suppressing glide phenomena.

In conclusion, this study investigates the structural evolution and phase transitions of Mn-based layered oxides for Na-ion battery (NIB) cathodes, specifically examining P2-Na_*x*_MnO_2_ (NMO) and its Ni-doped counterpart, P2-Na_*x*_Ni_0.25_Mn_0.75_O_2_ (NNMO). Using the VC-NEB method, we reveal the atomistic mechanisms behind the P2-to-P2′ and P2-to-OP4/O2 transitions across sodium levels, analyzing shifts in structural and electronic properties as the cathode operates. By breaking down structural distortions into Van Vleck modes at each charge state, we show that the transition mechanisms are driven by bond-length and bond-angle distortions during Mn/Ni oxidation. This crucial interplay determines the stability of the structure, underpinning the energy barriers that are involved. The three key findings from our study are the following.

(1) The high- and low-sodiation P2-to-P2′ and P2-to-OP4/O2 transitions are driven by two atomic movements: in-plane shifts of TMO_6_ octahedra and TMO_2_ layer gliding. The first affects the TM coordination shell, while the second prompts substantial stacking rearrangements. Both pathways reorganize the TM structure, leading to tetrahedral coordination in transition states.
(2) While the electronic structure is retained across the P-to-O transition, the conversion to P2′ upon resodiation leads to a higher concentration of Mn^3+^ and Ni^3+^. These JT active centers are responsible for bond-length distortions upon discharge and act as key drivers toward the low-voltage transition.
(3) The P-to-O glides are shown to be driven by shear distortions, which are effectively reduced by Ni, and even further by Li doping, suggesting that increasing TM-O covalency can improve the structural integrity upon charge.

Overall, our analysis highlights easily computable structural parameters that can act as descriptors for predicting the structural stability of TMO layered cathodes across charging/discharging NIB operations. By uncovering the driving forces and atomistic pathways behind undesired transitions, this work lays the foundations for further optimizing Na-based cathodes through strategic doping and structural tuning.

## Methods and Computational Details

First-principles calculations for P2-Na_*x*_MnO_2_ (NMO), P2-Na_*x*_Ni_0.25_Mn_0.75_O_2_ (NNMO), and P2-Na_*x*_Ni_0.125_Li_0.125_Mn_0.75_O_2_ (NNLMO) are performed using the projector augmented wave (PAW) method as implemented in the Vienna Ab Initio Simulation Package (VASP).^73−75^ Structure optimizations are conducted using the spin-polarized generalized gradient approximation (GGA) and employing the Perdew–Burke–Ernzerhof (PBE) exchange-correlation functional.^76^ To overcome the self-interaction error associated with DFT-GGA, especially for localized 3d electrons in transition metals, we use the DFT+U scheme with an effective on site correction parameter (U-J)_eff_ = 4.0 eV applied for both Mn and Ni d shells.^54^ The DFT-D3 method is employed with the Becke–Johnson (BJ) damping function to account for dispersive forces in these layered materials.^77−80^ A plane-wave energy cutoff of 750 eV and a 4 × 4 × 4 *k*-point grid based on the Monkhorst–Pack scheme are adopted, as determined from convergence tests.^81^ Electronic self-consistency convergence criteria for electronic minimization and ionic forces are set at 10^–5^ eV and 10^–3^ eV Å^–1^, respectively.

Structural models of the P2-NMO, P2-NNMO, and P2-NNLMO materials are constructed using 2√3 × 4 × 1 supercells, generated through orthogonal transformations of the original hexagonal unit cells.^42^ Depending on the sodium concentration (*x*_Na_), these supercells contain 120 to 100 atoms. To accurately describe the configurational entropy of Na distributions at each stoichiometry, the special quasi-random structure (SQS) approach is adopted by means of the SQSgen code.^82,83^ All structural parameters, including bond lengths and angular distortions, are calculated using the Van Vleck calculator.^66^ Transition pathways for the P2-to-P2′ and P2-to-OP4(O2) phase transformations in NMO and NNMO are obtained using the *pmpath* tool in the modified version developed by Samtsevich.^61^ We use √3 × 2 × 1 supercells for NMO and 2√3 × 4 × 1 supercells for NNMO to account for the lower sodium content. All direct comparisons between NMO and NNMO are performed using the 2√3 × 4 × 1 supercell to ensure consistency. The variable cell-nudged elastic band (VC-NEB) method^60^ is applied to all transition pathways using the USPEX interface,^61^ with the same optimization parameters as for structural relaxations. Vibrational frequencies and the dimer method are used on the maximum energy points along the P2-to-OP4 and P2-to-P2′ energy paths in NMO within the finite difference method implemented in VASP to verify the nature of the transition states (TS).^74,84^

## Supporting Information Available

VC-NEB validation, energy profiles for NNMO at different Na contents, crystal structures of OP4 and O2 phases, structural details of in-plane shifts and gliding motions, structural models for the transition states, electronic analyses along the transitions pathways, representations of Van Vleck modes and angular contributions, distortion parameters, cation distribution in P2-NNMO at *x*_Na_ = 0.25, pair distribution functions, angular distortions, and P2-NLNMO optimized structures (PDF)
