# Supplementary material for: Atomistic Insights into Solid-State Phase Transition Mechanisms of P2-Type Layered Mn Oxides for High-Energy Na-Ion Battery Cathodes
Source: ACS Energy Lett. 2025 Feb 6;10(3):1089–98. doi: 10.1021/acsenergylett.4c03335 (PMC11915757; doi:10.1021/acsenergylett.4c03335)
Supplement: Supplementary file 1 — nz4c03335_si_002.pdf [file nz4c03335_si_002.pdf]

SUPPORTING INFORMATION

Atomistic Insights into Solid-State Phase Transition Mechanisms of P2-Type Layered Mn Oxides for High-Energy Na-Ion Battery Cathodes

Aniello Langella,[a,b] Arianna Massaro,[a,b] Ana B. Muñoz-García,[b,c,*] Michele Pavone[a,b,*]

(a) *Department of Chemical Sciences, University of Naples Federico II, Complesso Univ. Monte Sant'Angelo Via Cintia 21, Naples 80126, Italy.*
(b) *National Interuniversity Consortium of Materials Science and Technology - Reference Center for Electrochemical Energy Storage (INSTM-GISEL), Via G. Giusti 9, Firenze 50121, Italy*
(c) *Department of Physics "E. Pancini", University of Naples Federico II, Complesso Univ. Monte Sant'Angelo Via Cintia 21, Naples, Italy.*

* Corresponding authors: ABMG anabelen.munozgarcia@unina.it; MP mipavone@unina.it

VC-NEB method validation

To validate our methodology, we assess phase stability prediction at different sodiation levels by focusing on P2-NMO structures at critical sodium contents of x_{Na} = 0.75 and x_{Na} = 0.375 as starting points. For both x_{Na}, we analyze both the P2-to-P2' and P2-to-OP4 transitions and determine the resulting energy barriers. As shown on the right side of **Figure S1**, the P2-P2' transition (blue line) exhibits a lower energy barrier than the P2-to-OP4 one (red line) at high sodiation degree (1.24 eV *vs.* 1.94 eV, respectively). Conversely, on the left side of **Figure S1**, the P2-to-OP4 transition (red line) is energetically favored compared to the P2-to-P2' (blue line) at low sodium content, with energy barriers of 1.37 eV *vs.* 1.62 eV. These results consistently align with the experimental observation of P2' and OP4 phases formed at, respectively, high and low sodium concentration over the initial P2 phase.[1,2]

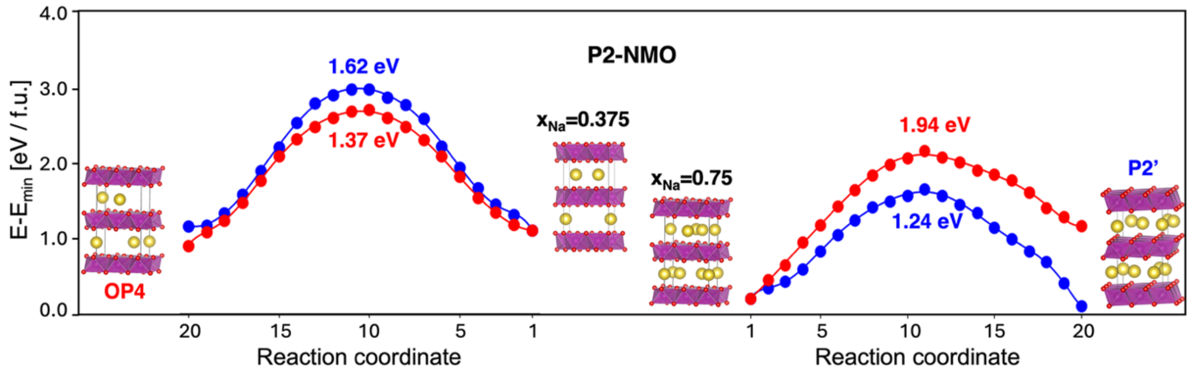

Figure S1 *Energy profiles for phase transitions at critical Na content x_{Na} in NMO. P2-to-P2' and P2-to-O transitions and corresponding barriers are plotted in blue and red, respectively. Color code as in Figure 1.*

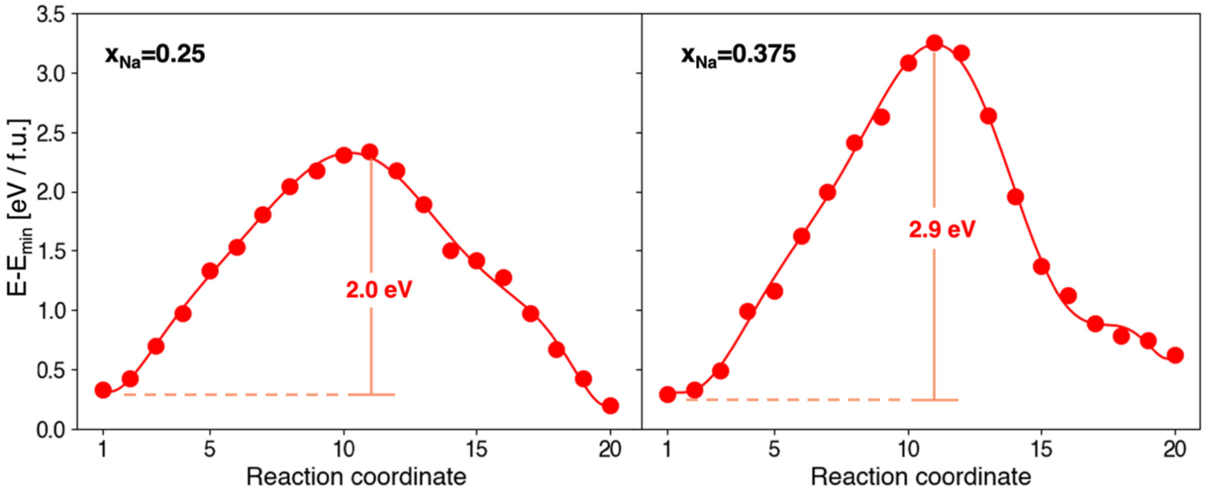

Figure S2. *Energy profiles for the P2-to-O2 transition in NNMO at x_{Na} = 0.25(**left**) and x_{Na} = 0.375, (**right**) respectively.*

Crystal structure differences in OP4- and O2- materials

The OP4- and O2-layered transition metal oxides share the same space group, $P6_3mc$ (no. 186),[1,3] where each intermediate layer of the TMO_6 framework can, in theory, accommodate sodium either in prismatic sites (Wyckoff positions 2d and 2c) or in octahedral sites (Wyckoff position 2a).[1] However, experimental evidence shows that these structures cannot support both octahedral and prismatic sodium coordinations within the same layer.[4] In the OP4 structure, sodium alternates between prismatic and octahedral sites in adjacent layers, while in the O2 structure, sodium exclusively occupies octahedral sites.[1,5] This subtle structural difference results in a striking effect: the OP4 phase exhibits a pronounced expansion along the c-axis, whereas the O2 phase, which contains only octahedral sodium, shows a contraction of the c-axis relative to the P2 phase.[1,5,6]

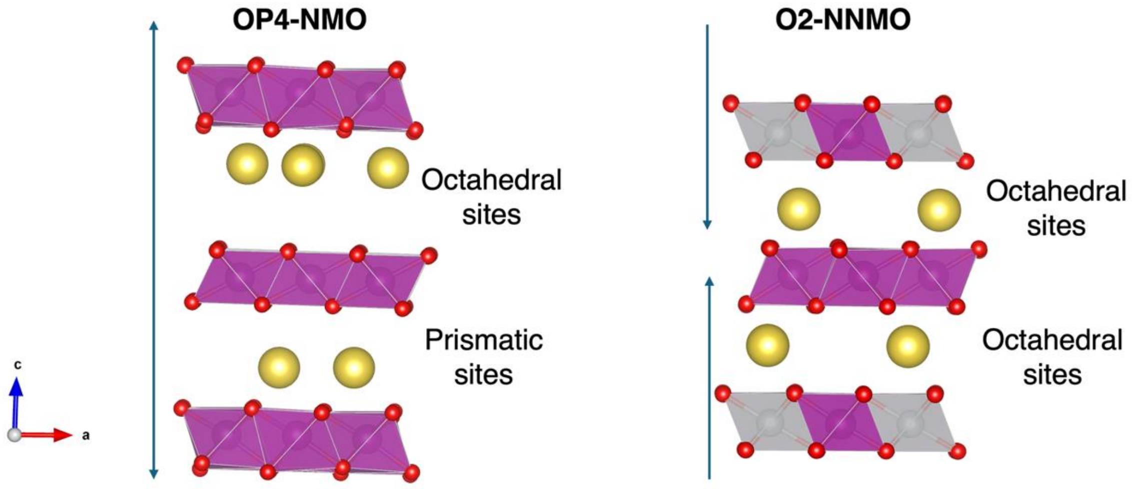

Figure S3. *Different sodium coordination in OP4 (**left**) and O2 (**right**) materials. Arrows indicate expansion relative to the P2 phase for OP4 and contraction for O2. Color code as in Figure 1.*

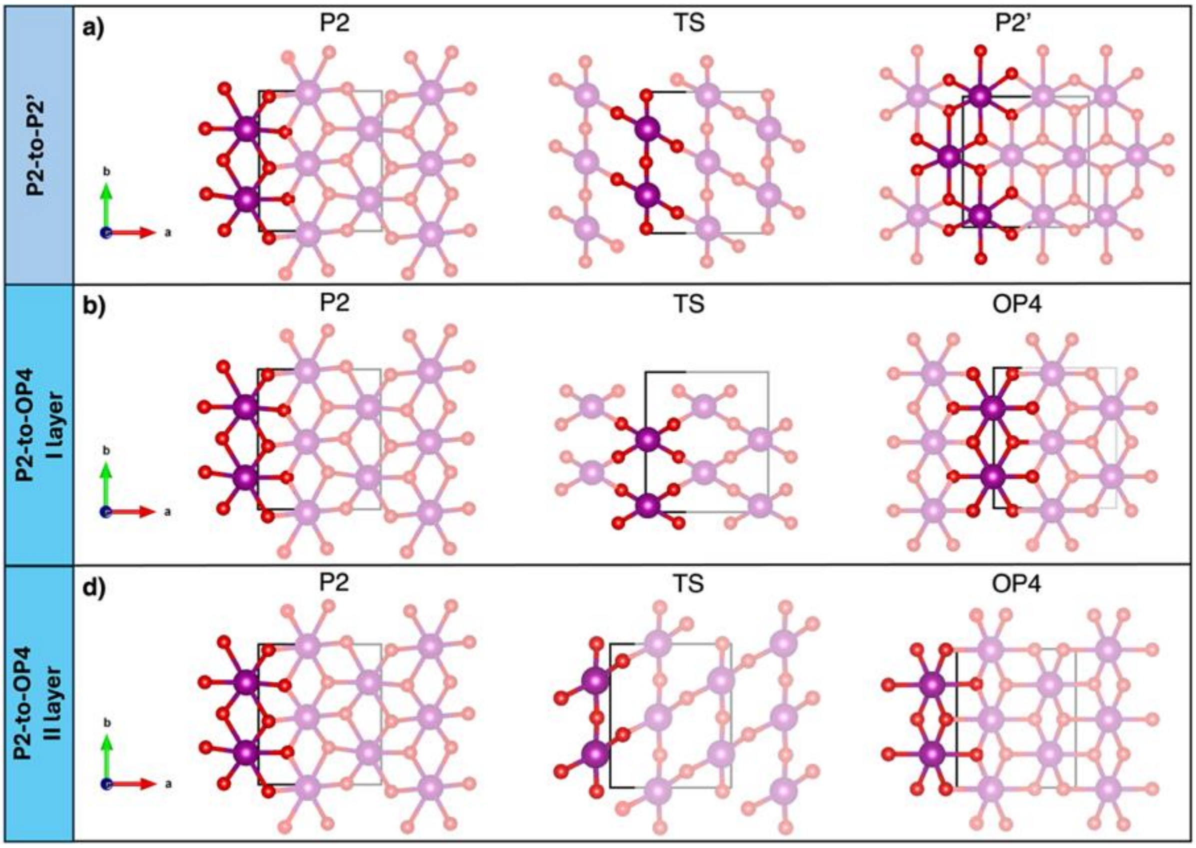

Figure S4. *In-plane shifts in P2-NMO during (**a**) the P2-to-P2' transition and (**b-c**) the P2-to-OP4 transition. **(a)**In the P2-to-P2' transformation, all layers experience the same shift pattern. **(b-c)** In the P2-to-OP4 transition, adjacent layers undergo distinct shifts, with one layer shifting forward and the neighboring layer moving backward. These shifts are highlighted by keeping only a single row of atoms fully opaque. Color code as in Figure 1.*

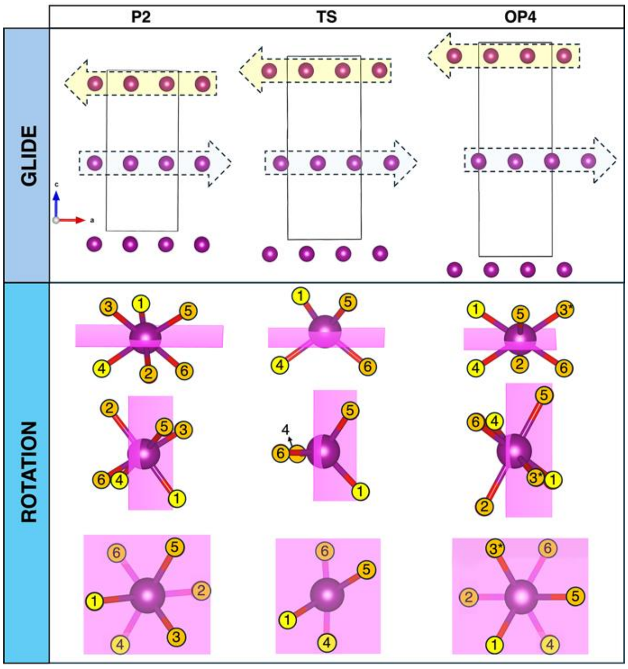

Figure S5. (Top) *Glide motion in P2-NMO during P2-to-OP4 phase transitions, showing only Mn atoms for a better visualization.* ***(Bottom)*** *Rotation motion in P2-NMO during P2-to-OP4 transition, showing only one Mn center and varying perspectives of ab-plane depicted in purple. For atom color refers to Figure 1.*

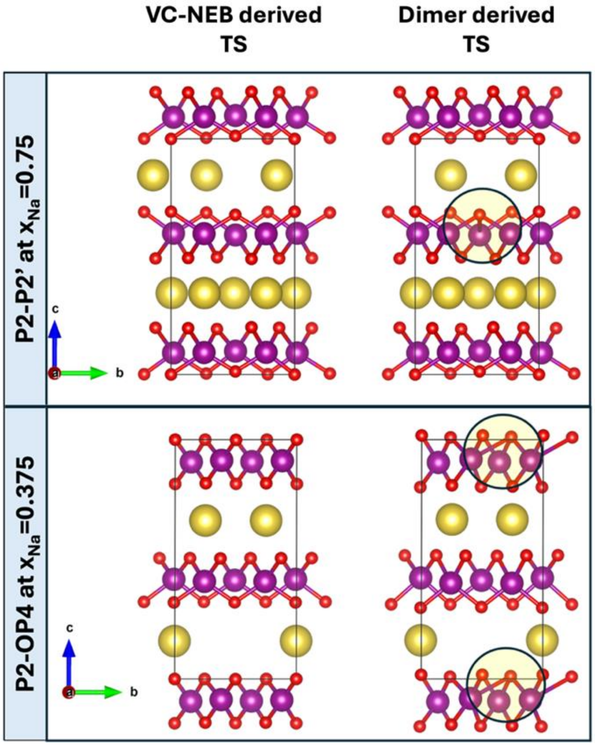

Figure S6. *Comparison of the TS along the P2-to-P2' and the P2-to-OP4 transitions as derived from the VC-NEB method and the standard dimer method.*

Electronic structure analysis

The electronic properties of P2-NMO and P2-NNMO phases during battery operation have been widely studied, providing valuable insights into the behavior of these materials under varying sodium content.[2,7] Here, we focus on electronic changes at critical sodium contents associated with phase transitions—specifically, x_{Na} = 0.75 for the P2-to-P2' transition in both NMO and NNMO, and x_{Na} = 0.375 and 0.125 for the P-O transition in NMO and NNMO, respectively. Our analysis, which includes projected density of states (PDOS), net magnetic moments and Bader charge assessments to clarify the factors driving these transitions.

In the P2-to-P2' transition in NMO (**Figure SI 7**), we observe an increase in the manganese net magnetic moment, which is accompanied by a depletion of the manganese d-band (shown in light purple in the PDOS) just above the Fermi level and increased occupancy just below it (see **SI 7 a, b**). This rise in Mn magnetic moment indicates a partial reduction of manganese (from Mn^{4+} to Mn^{3+}), confirmed by Bader charge analysis (**SI 7 c**), which shows a trend toward lower manganese charge. Similarly, in NNMO (**Figure SI 9**) the P2-to-P2' transition, also involves partial manganese reduction, as seen in the Mn net magnetic moment and in PDOS (**SI 9 a, b**). Concurrently, the magnetic moment of nickel decreases (**SI 9 c**), indicating its partial oxidation (from Ni^{2+} to Ni^{3+}). This oxidation is evident in the PDOS as increased population in the nickel d-band (in light gray) near the Fermi energy, resembling electronic features reported for fully lithiated LNO.[8] This band occupancy near the Fermi level corresponds to the Ni d_{z^2}orbital, indicating the presence of Ni^{3+}.

For the P2-to-OP4 transition in NMO (**Figure SI 8**), a milder partial reduction of manganese is observed. Similarly, in NNMO (**Figure SI 10**), both manganese and nickel exhibit slight reductions during the transition. Notably, the density of states (DOS) for the O2 and OP4 phases seem to differ significantly, likely due to structural variations, as shown in Figure SI 3. Specifically, the OP4 phase is expanded along c-axis compared to P2 structure, while the O2 structure is contracted in this direction. Qualitatively, the PDOS for OP4 can be viewed as an enlarged form of the PDOS for O2 and, conversely, the PDOS of O2 as the contracted form of OP4.

This electronic structure analysis reveals that the P2-to-P2' transition is primarily influenced by the presence and increase of Jahn-Teller-active centers, like Mn^{3+} a Ni^{3+}. Additionally, the tetrahedral transition state in NNMO is closely associated with Ni^{3+}, as observed in the P2-to-O2 transition (SI 10), where the reduction from Ni^{4+} to Ni^{3+} (P2 to TS) is clear in the nickel magnetic moments. This shift is consistent with the d^7 configuration of Ni^{3+}, which readily supports tetrahedral coordination.

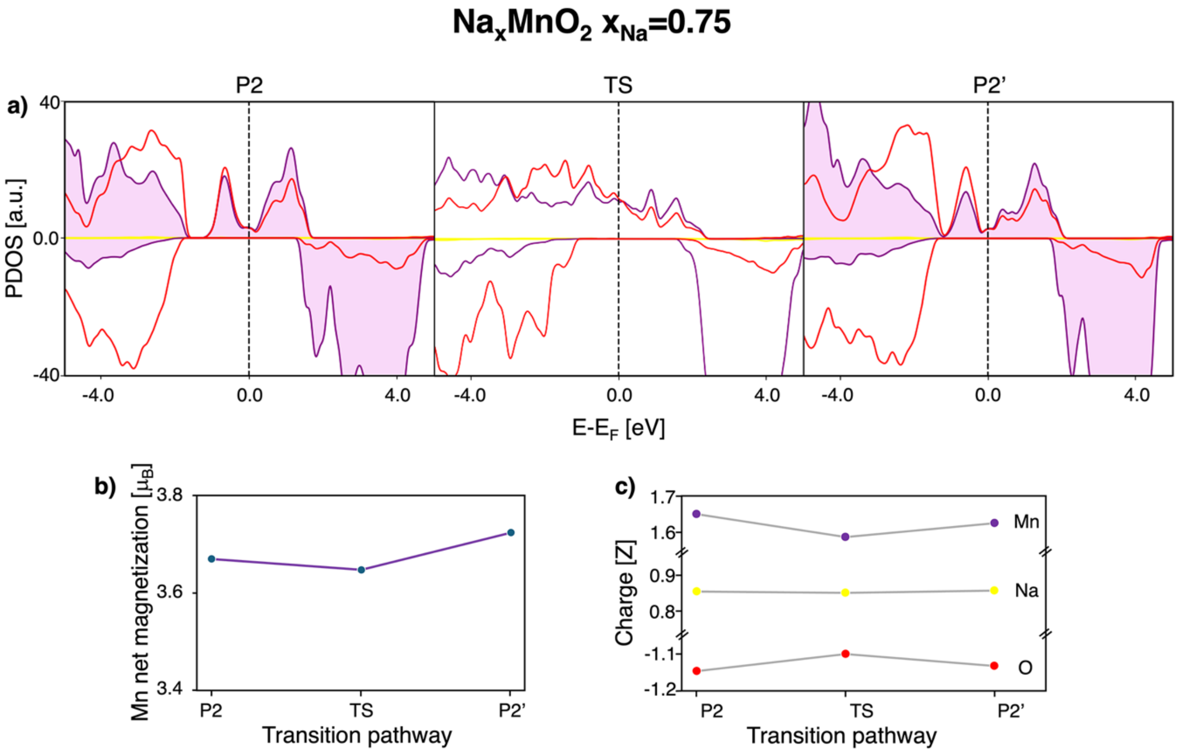

***Figure S7. (a)** Atom-, angular momentum-, and spin-projected density of states (PDOS) for P2-to-P2' transition in $Na_{0.75}MnO_2$ computed at the PBE+U-D3(BJ) level of theory. Color code: Na s states, yellow; Mn d states, violet; O p states, red. **(b)** Net magnetization of Mn atoms plotted for P2-to-P2' transition in $Na_{0.75}MnO_2$ as computed at the PBE+U-D3(BJ) level of theory. **(c)** Bader charge analysis reported as the average charge for each element.*

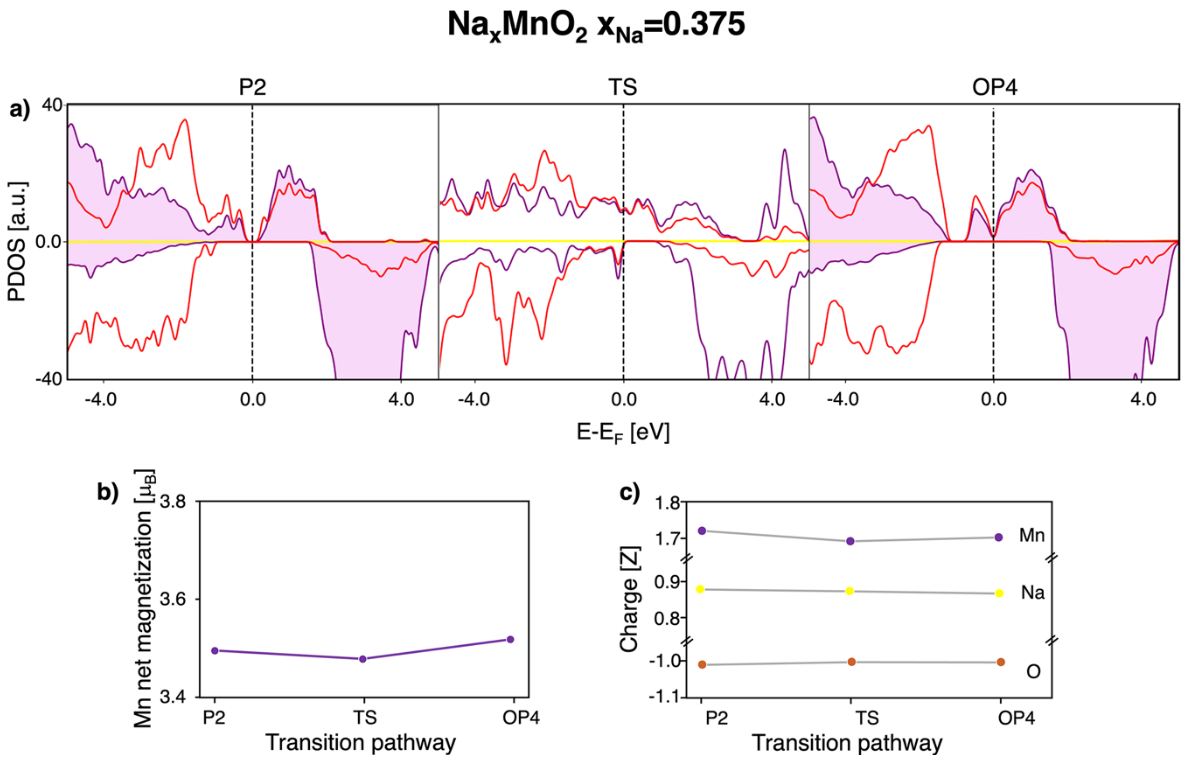

Figure S8. (a) *Atom-, angular momentum-, and spin-projected density of states (PDOS) for P2-to-OP4 transition in $Na_{0.375}MnO_2$ computed at the PBE+U-D3(BJ) level of theory. Colour code: Na s states, yellow; Mn d states, violet; O p states, red.* ***(b)*** *Net magnetization of Mn atoms plotted for P2-to-OP4 transition in $Na_{0.375}MnO_2$ as computed at the PBE+U-D3(BJ) level of theory.* ***(c)*** *Bader charge analysis reported as the average charge for each element.*

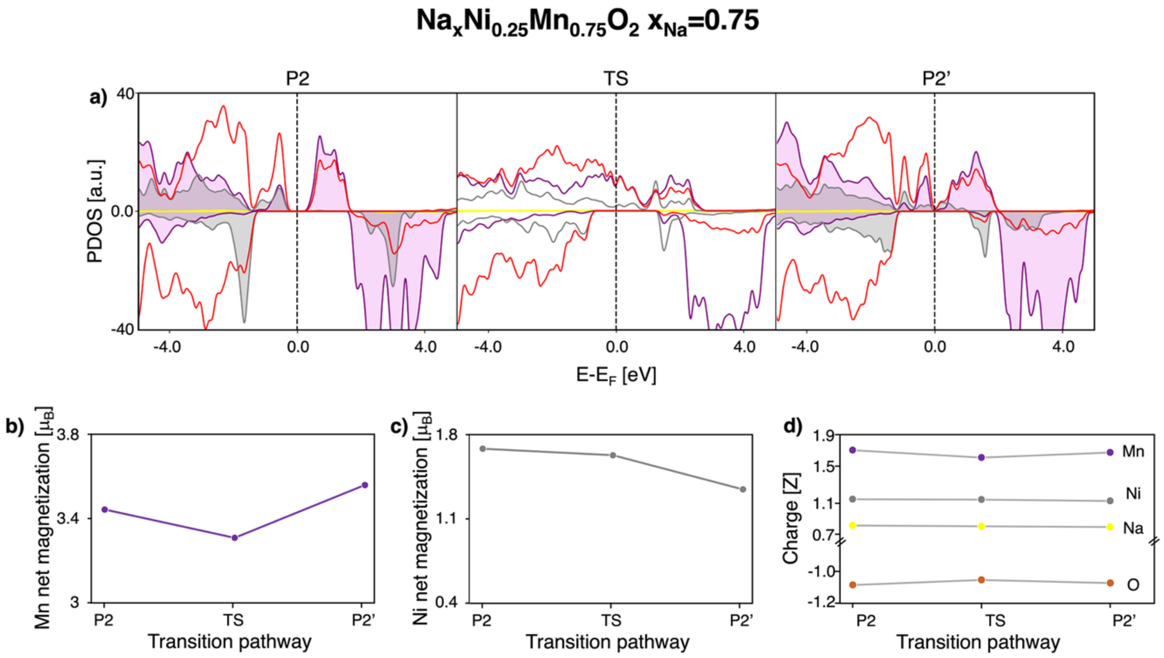

Figure S9. ***(a)*** *Atom-, angular momentum-, and spin-projected density of states (PDOS) for P2-to-P2' transition in* $Na_{0.75}Ni_{0.25}Mn_{0.75}O_2$ *computed at the PBE+U-D3(BJ) level of theory. Colour code: Na s states, yellow; Ni d states, gray; Mn d states, violet; O p states, red.* ***(b, c)*** *Net magnetization of Mn (purple) and Nickel (grey) atoms plotted for P2-to-P2' transition in* $Na_{0.75}Ni_{0.25}Mn_{0.75}O_2$ *as computed at the PBE+U-D3(BJ) level of theory.* ***(d)*** *Bader charge analysis reported as the average charge for each element.*

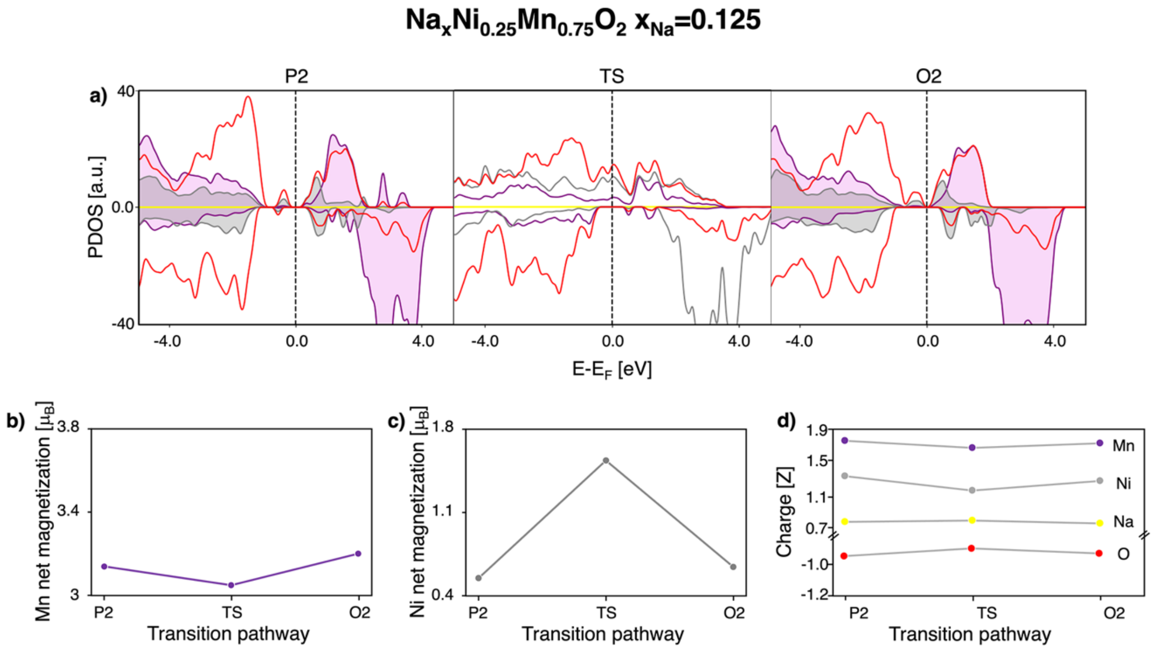

Figure S10. ***(a)*** *Atom-, angular momentum-, and spin-projected density of states (PDOS) for P2-to-O2 transition in* $Na_{0.125}Ni_{0.25}Mn_{0.75}O_2$ *computed at the PBE+U-D3(BJ) level of theory. Colour code: Na s states, yellow; Ni d states, gray; Mn d states, violet; O p states, red.* ***(b, c)*** *Net magnetization of Mn (purple) and Nickel (grey) atoms plotted for P2-to-O2 transition in* $Na_{0.75}Ni_{0.125}Mn_{0.75}O_2$ *as computed at the PBE+U-D3(BJ) level of theory.* ***(d)*** *Bader charge analysis reported as the average charge for each element.*

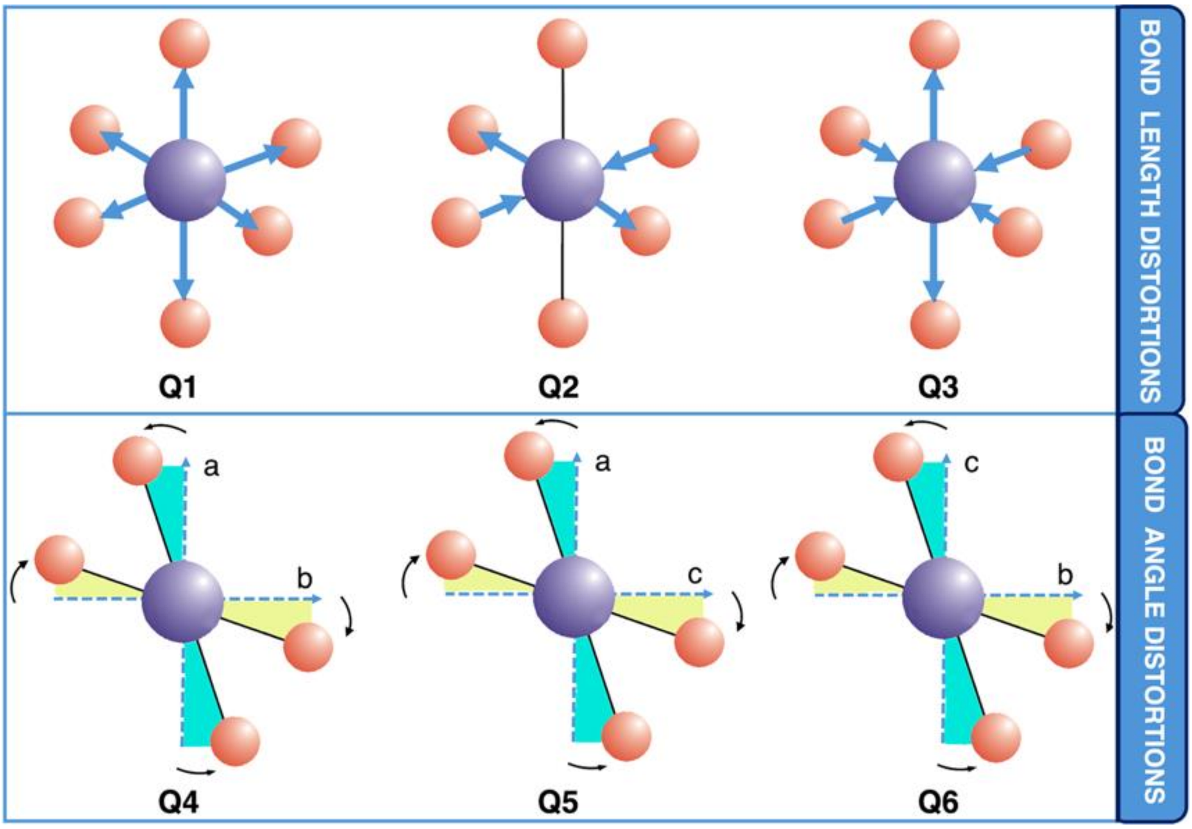

Figure S11. *Graphical representation of Van Vleck modes in an octahedron:* ***(top)*** *bond length distortions (Q1, Q2, and Q3), including elongation or compression, are highlighted by blue arrows;* ***(bottom)*** *bond angle distortions (Q4, Q5, and Q6), including shear modes, are illustrated by curved black arrows and yellow/cyan regions.*

Distortion parameters

The primary source of distortions in layered oxide structures is typically attributed to the Jahn-Teller effect,[3-5] which affects TM-O bond distances. Various methods are proposed in the literature to parameterize JT distortion, usually based on bond distance variations,[9] effective coordination number,[10] or volumetric changes.[11] Here, to quantify these distortions in TMO_6 layers, we use the average octahedral distortion, D_{OCT}, comparable to the bond-length distortion index (BLDI) defined by Bauer,[9] as shown in Equation (1):[12]

$$\%D_{oct} = \left(\frac{6(l_{long}-l_{short})}{2l_{long}+4l_{short}}\right)\cdot 100 \quad (1)$$

where l_{long} and l_{short} represent the elongated and ideal TM-O bond lengths, respectively. Given the predominance of manganese in the structures analyzed, a threshold of 1.94 Å was used for l_{long} and l_{short}, corresponding to the average Mn–O bond length in an ideally undistorted MnO_6 octahedron.[13] While the Jahn-Teller effect is undoubtedly a source of distortion, it is also important to recognize that octahedra, though often modeled as rigid bodies, are not fully rigid in many systems.[14] Octahedral tilting frequently induces strain, resulting in angular distortions. To better understand the relative impact of the Jahn-Teller effect versus tilting, we evaluated Van Vleck modes using the Van Vleck Calculator.[14] The average magnitude of the distortion, ρ_0, is defined as:

$$\rho_0 = (Q_2^2 + Q_3^2)^{1/2}$$

where Q2 and Q3 are modes sensitive to bond-length distortions directly associated with the Jahn-Teller effect. Meanwhile, angular distortions are quantified with the Q4 to Q6 modes. Another factor are the shear modes, which indicate whether opposing ligands move in the same or opposite direction (**Figure S12**). An octahedron may have angular distortion (non-zero Q4 to Q6 modes) without exhibiting octahedral shear. To further understand shear modes, we use the fraction parameter, h, proposed by Nagle-Cocco and Dutton:[14]

$$\eta = \frac{\Delta^2_{\text{shear}}}{\Delta^2_{\text{shear}}-\Delta^2_{\text{anti-shear}}} \quad (3)$$

where D_{shear} and $D_{anti\text{-}shear}$ measure angular variations across different planes of the octahedron and are intrinsically related to the Q4, Q5 and Q6 modes.[14]

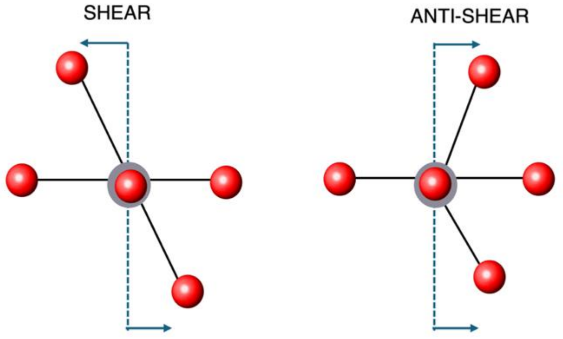

Figure S12. *Schematic representation of shear and anti-shear modes observed in the ab plane of an octahedron.*

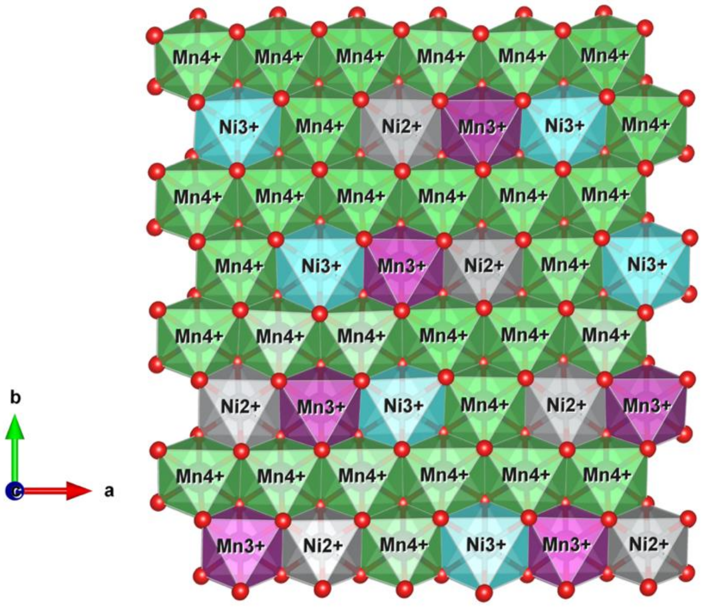

Figure S13. *Cations distribution in the ab-plane of P2-NNMO lattice at* x_{Na} *= 0.25. Na atoms are removed for clarity. Color code:* Mn^{3+}*-centered octahedra, purple;* Mn^{4+}*-centered octahedra, green.* Ni^{2+}*-centered octahedra, gray;* Ni^{3+}*-centered octahedra, turquoise.*

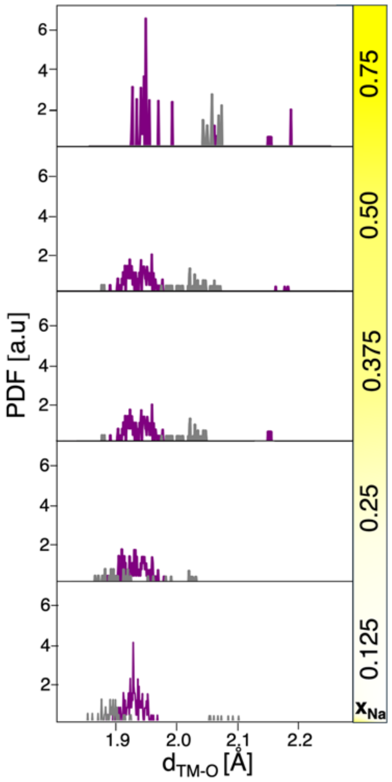

Figure S14. *Pair distribution functions (PDFs) of the Mn-O (purple) and Ni-O (gray) bond distances in P2-NNMO at different Na contents.*

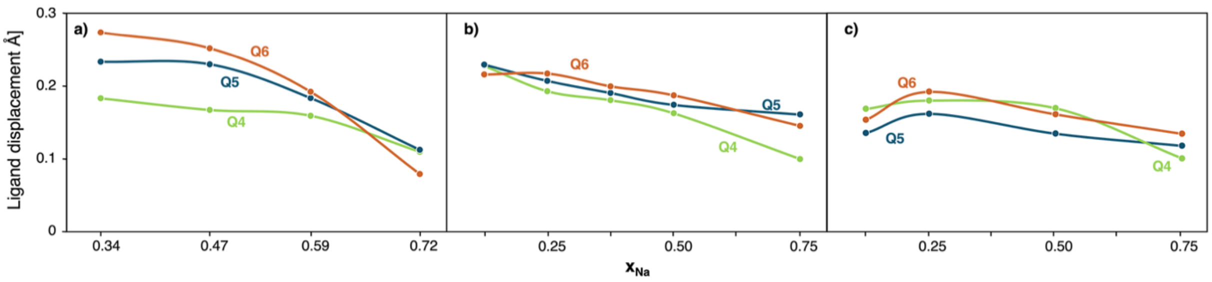

Figure S15. *Averaged Van Vleck modes (Q4 to Q6) for each TMO_6 octahedron in P2-NMO (**a**), P2-NNMO (**b**) and P2-NLNMO (**c**) plotted as a function of x_{Na}. All values were calculated using the Van Vleck calculator[66]**.***

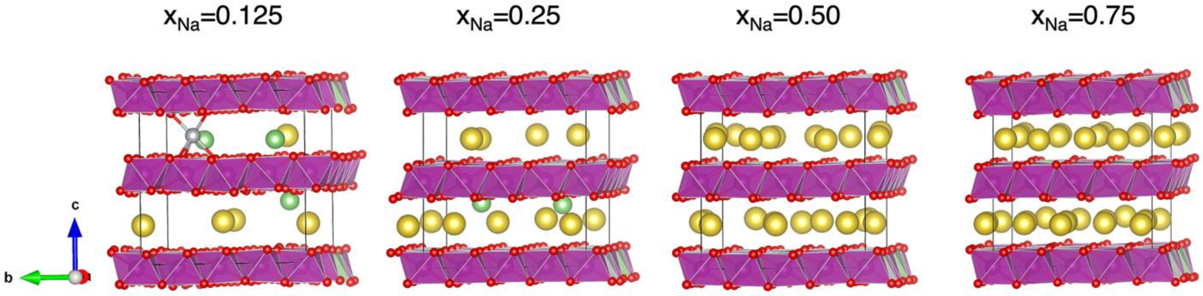

Figure S16. *P2-NLNMO structures optimized at the PBE+U-D3(BJ) level of theory. Color code: Na, yellow; Mn, violet; O, red; Ni, gray; Li, green.*

Supplementary references

1. Wang, C.; Liu, L.; Zhao, S.; Liu, Y.; Yang, Y.; Yu, H.; Lee, S.; Lee, G.-H.; Kang, Y.-M.; Liu, R.; Li, F.; Chen, J. Tuning Local Chemistry of P2 Layered-Oxide Cathode for High Energy and Long Cycles of Sodium-Ion Battery. *Nat. Commun.* **2021**, *12*, 2256. https://doi.org/10.1038/s41467-021-22523-3.
2. Langella, A.; Massaro, A.; Muñoz-García, A. B.; Pavone, M. First-Principles Insights on Solid-State Phase Transitions in P2- Na_xMnO_2-Based High Energy Cathode during Na-Ion Battery Operations. *Chem. Mater.* **2024**, 36, 2370–2379. https://doi.org/10.1021/acs.chemmater.3c02981.
3. Lee, D. H.; Xu, J.; Meng, Y. S. An Advanced Cathode for Na-Ion Batteries with High Rate and Excellent Structural Stability. *Phys. Chem. Chem. Phys.* **2012**, *15*, 3304. https://doi.org/10.1039/c2cp44467d.
4. Wang, P.; You, Y.; Yin, Y.; Wang, Y.; Wan, L.; Gu, L.; Guo, Y. Suppressing the P2–O2 Phase Transition of Na0.67Mn0.67Ni0.33O2 by Magnesium Substitution for Improved Sodium-Ion Batteries. *Angew. Chem., Int. Ed.* **2016**, *55*, 7445–7449. https://doi.org/10.1002/anie.201602202.
5. Wang, P.; You, Y.; Yin, Y.; Guo, Y. Layered Oxide Cathodes for Sodium-Ion Batteries: Phase Transition, Air Stability, and Performance. *Adv. Energy Mater.* **2017**, *8*, 1701912. https://doi.org/10.1002/aenm.201701912.
6. Hou, P.; Lin, Z.; Dong, M.; Sun, Z.; Gong, M.; Li, F.; Xu, X. A Thermodynamically Stable O2-Type Cathode with Reversible O2-P2 Phase Transition for Advanced Sodium-Ion Batteries. *J. Colloid. Interface Sci.* **2023**, *649*, 1006–1013. https://doi.org/10.1016/j.jcis.2023.06.162.
7. Massaro, A.; Muñoz-García, A. B.; Prosini, P. P.; Gerbaldi, C.; Pavone, M. Unveiling Oxygen Redox Activity in P2-Type $Na_xNi_{0.25}Mn_{0.68}O_2$ High-Energy Cathode for Na-Ion Batteries. *ACS Energy Lett.* **2021**, *6*, 2470–2480. https://doi.org/10.1021/acsenergylett.1c01020.
8. Yousuf, S.; Mridha, M. M.; Magri, R. Structures and Electronic States of Nickel-Rich Oxides for Lithium Ion Batteries. *Mat. Adv.* **2024**, *5*, 2069–2087. https://doi.org/10.1039/d3ma00906h.
9. Baur, W. H. The Geometry of Polyhedral Distortions. Predictive Relationships for the Phosphate Group. Acta *Acta Crystallogr. B* **1974**, *30*, 1195–1215. https://doi.org/10.1107/s0567740874004560.
10. Hoppe, R. Effective Coordination Numbers (ECoN) and Mean Fictive Ionic Radii (MEFIR). *Z. Kristallogr. Cryst. Mater.* **1979**, *150*, 23–52. https://doi.org/10.1524/zkri.1979.150.14.23.
11. Robinson, K.; Gibbs, G. V.; Ribbe, P. H. Quadratic Elongation: A Quantitative Measure of Distortion in Coordination Polyhedra. *Science*, **1971**, *172*, 567–570. https://doi.org/10.1126/science.172.3983.567.
12. Jung, H.; Kim, J.; Kim, S. Phonon Study of Jahn–Teller Distortion and Phase Stability in $NaMnO_2$ for Sodium-Ion Batteries. *J. Appl. Phys.* **2022**, *132*, 055101 https://doi.org/10.1063/5.0086903.
13. Wang, P.-F.; Jin, T.; Zhang, J.; Wang, Q.-C.; Ji, X.; Cui, C.; Piao, N.; Liu, S.; Xu, J.; Yang, X.-Q.; Wang, C. Elucidation of the Jahn-Teller Effect in a Pair of Sodium Isomer. *Nano Energy*, **2020**, *77*, 105167. https://doi.org/10.1016/j.nanoen.2020.105167.
14. Nagle-Cocco, Liam. A. V.; Dutton, S. E. Van Vleck Analysis of Angularly Distorted

Octahedra Using VanVleckCalculator. *J. Appl. Crystallogr.*, **2024**, 57, 20–33. https://doi.org/10.1107/s1600576723009925.
